# Eco‐evolutionary dynamics driven by fishing: From single species models to dynamic evolution within complex food webs

**DOI:** 10.1111/eva.13058

**Published:** 2020-08-26

**Authors:** Tommi Perälä, Anna Kuparinen

**Affiliations:** ^1^ Department of Biological and Environmental Sciences University of Jyväskylä Jyväskylä Finland

**Keywords:** aquatic ecosystems, co‐evolution, community dynamics, ecosystem dynamics, Eurasian perch, food webs, lake constance, life‐history evolution, predator‐prey dynamics

## Abstract

Evidence of contemporary evolution across ecological time scales stimulated research on the eco‐evolutionary dynamics of natural populations. Aquatic systems provide a good setting to study eco‐evolutionary dynamics owing to a wealth of long‐term monitoring data and the detected trends in fish life‐history traits across intensively harvested marine and freshwater systems. In the present study, we focus on modelling approaches to simulate eco‐evolutionary dynamics of fishes and their ecosystems. Firstly, we review the development of modelling from single species to multispecies approaches. Secondly, we advance the current state‐of‐the‐art methodology by implementing evolution of life‐history traits of a top predator into the context of complex food web dynamics as described by the allometric trophic network (ATN) framework. The functioning of our newly developed eco‐evolutionary ATNE framework is illustrated using a well‐studied lake food web. Our simulations show how both natural selection arising from feeding interactions and size‐selective fishing cause evolutionary changes in the top predator and how those feed back to its prey species and further cascade down to lower trophic levels. Finally, we discuss future directions, particularly the need to integrate genomic discoveries into eco‐evolutionary projections.

## 1 INTRODUCTION

The study of eco‐evolutionary dynamics has become a prominent field in the contemporary biology research (Hendry, 2017). The field and its current terminology was largely catalysed by the realization that evolution occurs more rapidly when viewed across contemporary time scales (Hendry & Kinnison, 1999), yet its roots stem from the earlier studies looking into the role of trait evolution on species interactions such as consumer–resource and host–parasite dynamics (Abrams & Matsuda, 1997; Pimentel, 1961; Pimentel & Al‐Hafidh, 1965; van Valen, 1973). As shown by these and more recent studies, the stability (Hiltunen, Hairston, Hooker, Jones, & Ellner, 2014) and the ecological outcome of the interactions (Kasada, Yamamichi, & Yoshida, 2014) can be highly dependent on genetic variation and trade‐offs associated with key fitness‐related traits. From a more applied perspective, the potential for eco‐evolutionary dynamics implies that anthropogenic selection and changes in mortality regimes can cause evolutionary shifts and demographic changes in natural populations over just a few generations (e.g. Coltman et al., 2003; Law & Grey, 1989; Stearns, 1983).

In the aquatic context, the transplantation experiment of Trinidadian guppies (*Poecilia reticulata*) was one of the seminal studies illustrating rapid evolution in response to environmental conditions: guppies transplanted from high to low predation environments evolved towards later maturity and lower reproductive investment, which matched the phenotypes that occurred naturally in low predation environments (Reznick, Shaw, Rodd, & Shaw, 1997). These phenotypic changes further extended to ecosystem‐level processes (Bassar et al., 2010; El‐Sabaawi, Bassar, et al., 2015). Similarly, five generations of directional selection on body size in the common garden experiment caused Atlantic silverside (*Menidia menidia*) body size to evolve either larger or smaller with respect to the initial body sizes depending on the selection regime (Conover & Munch, 2002; Therkildsen et al., 2019). The body size changes observed in silverside were further associated with correlated traits such as behaviour and reproduction (Walsh, Much, Chiba, & Conover, 2006). These examples suggest that anthropogenic impacts as well as changes in aquatic community structure may drive relatively rapid evolutionary changes in species and, in turn, affect their ecological properties in single and multispecies levels.

Aquatic systems provide in many ways a good setting to study eco‐evolutionary dynamics. Firstly, many aquatic species, particularly fishes, harbour a much larger amount of phenotypic diversity than terrestrial species (Kuparinen & Festa‐Bianche, 2017). Secondly, being ectotherms and indeterminate growers, mean phenotypes and phenotypic variability of aquatic species can be easily modified by temperature and food availability (Charnov, 1993; O'Dea, Lagisz, Hendry, & Nakagawa, 2019). On the other hand, because of projected changes in these environmental drivers coupled with intensive and size‐selective fishing, aquatic organisms are likely to experience strong selective pressures on their key fitness‐related traits, such as growth, body size, the timing of maturity and reproductive investment (Cheung et al., 2013; Swain, Sinclair, & Hanson, 2007). Two other reasons to study eco‐evolutionary dynamics in aquatic systems are that there are (a) considerable amounts of data collected by fisheries‐independent surveys and landing records and (b) well‐developed statistical methods to assess ecological properties of fish populations, such as the census population size, the rate of reproduction, density dependence and the rates on natural and total mortalities (e.g. Hilborn & Walters, 1992). More recently, new genomic methods are entering the tool box of fisheries scientists such that the identification of the boundaries of natural populations, quantification of gene flow and detection of changes in the gene frequencies can provide novel insights into the evolutionary processes and their drivers (Bernatchez et al., 2017; Kuparinen & Hutchings, 2019).

Detailed information about the demographic rates governing fish populations has facilitated the development of mechanistic simulation approaches to study ecological and evolutionary processes in fishes (e.g. Dunlop, Heino, & Dieckmann, 2009). In the present study, we focus on modelling attempts to investigate and predict eco‐evolutionary dynamics in aquatic systems with a particular focus on fishing as the key driver [for a review on experimental approaches on eco‐evolutionary dynamics in aquatic systems, see, for example De Meester and Pantelj (2014)]. Firstly, we review the development of modelling approaches from single species to multispecies systems with increasing ecological complexity. Secondly, we take the first steps to advance the state‐of‐the‐art by introducing a novel way to model eco‐evolutionary dynamics in the context of complex aquatic food webs. We describe the theoretical basis of the approach as well as illustrate its functioning in the empirically parameterized Lake Constance food web (Boit, Martinez, Williams, & Gaedke, 2012; Kuparinen, Boit, Valdovinos, Lassaux, & Martinez, 2016; Kuparinen, Perälä, Martinez, & Valdovinos, 2019).

## 2 MERGING GENETICS INTO THE MODELS OF FISH POPULATION DYNAMICS

The first attempts to simulate eco‐evolutionary dynamics in aquatic species stemmed from the observation that many commercially harvested fish populations showed a declining trend in body size and age at maturation (e.g. Hutchings & Baum, 2005; Law, 2000) as well as in their probabilistic maturation reaction norms (Devine, Wright, Pardoe, & Heino, 2012). Inspired by the advances in “adaptive dynamics,” several so‐called eco‐genetic models for population dynamics were developed (reviewed by Dunlop et al., 2009). The idea of these models was that individual‐based population dynamics involved a component that mimicked inheritance of one or several life‐history traits (Dunlop et al., 2009; Enberg, Jørgensen, Dunlop, Heino, & Dieckmann, 2009). In practice, the traits of the juveniles reflected the average across the parents, coupled with some normally distributed plasticity to match with the assumed trait heritability. Models like this were then used to simulate how selective fishing might alter life‐history traits of the individuals (Jørgensen, Ernande, & Fiksen, 2009) and how changes in fish life histories can alter the ecological dynamics of the population, for example, its recovery ability (Enberg et al., 2009), density‐dependence of reproduction (Enberg, Jørgensen, & Mangel, 2010), natural mortality (Jørgensen & Fiksen, 2010) and fisheries yields (Eikeset et al., 2016; Enberg et al., 2009).

One of the key limitations of the first eco‐genetic models was their inordinately high number of free parameters. Another limitation, which was identified by Wang and Höök (2009), was the assumption that stems from the quantitative genetic basis of the inheritance of life‐history traits: the assumption that traits are normally distributed. In response to these disadvantages, alternative types of single species eco‐evolutionary models were developed based on the principles of Mendelian inheritance (Kuparinen, Hardie, & Hutchings, 2012; Wang & Höök, 2009) as well as on empirically observed life‐history invariants and growth along von Bertalanffy curves (von Bertalanffy, 1938; Kindsvater & Palkovacs, 2017; Kuparinen et al., 2012). Similar to the first eco‐genetic models, these approaches were applied to simulate eco‐evolutionary dynamics of fish populations exposed to alternative fishing regimes (Kuparinen et al., 2012; Kuparinen & Hutchings, 2012; Wang & Höök, 2009). Also, the emerging patterns of eco‐evolutionary dynamics driven by fishing were fairly similar: intensive, potentially size‐selective fishing leads to decreases in the age and size at maturity (e.g. Enberg et al., 2009; Kuparinen & Hutchings, 2012) as well as increased natural mortality through the survival costs of reproduction (Jørgensen & Fiksen, 2010). These effects reduce population equilibrium abundance, such that the phenotypic recovery is relatively slow even in the absence of fishing (e.g. Enberg et al., 2009). The utility of alternative formulations largely depends on the study question and data availability as critically reviewed by Dunlop et al. (2009).

The most notable differences in the nature of fishing‐driven eco‐evolutionary dynamics have been predicted to result from the genetic architecture underlying phenotypic variability. While traditionally it has been assumed that quantitative traits are coded by a large number of loci with small additive effects (Roff, 2002), in Atlantic salmon (*Salmo salar*) the age at maturity was recently discovered to be strongly controlled by only one locus with sexually dimorphic expression (Barson et al., 2015). The eco‐evolutionary dynamics resulting from this trait architecture dramatically differ from those predicted by traditional quantitative genetics: while the latter leads to directional phenotypic change and a reduction in phenotypic diversity, the former causes disruptive and divergent evolution that further feeds back to increased stochasticity in the intrinsic per capita population growth (Kuparinen & Hutchings, 2017). Nonetheless, investigations about the role of genetic architecture on eco‐evolutionary dynamics remain limited. Only few phenotype‐coding “supergenes” or “keystone genes” have been identified so far, although their frequency has been speculated to be substantial (Skovmand et al., 2018; Thompson & Jiggins, 2014; Wellenreuther & Bernatchez, 2018). For example, in Chinook salmon migration timing was recently found to be tightly linked with one single locus (Thompson et al., 2019).

## 3 INCREASED ECOLOGICAL REALISM: CHANGING FISH LIFE HISTORIES WITHIN ECOSYSTEMS

The above discussed investigations of eco‐evolutionary dynamics in aquatic systems suffer from one major limitation: they are limited to single species dynamics and, thus, lack the ecological realism arising from multispecies interactions. Namely, evolution driven by fishing can modify the species' trophic position and thereby likely affect predator‐prey interactions and food web dynamics (Kindsvater & Palkovacs, 2017). The need for multispecies approaches is further supported by several empirical approaches and theoretical analyses of predator–prey dynamics: predators can directly or indirectly through trait‐mediated changes cause cascades throughout food webs (Fahimipour, Anderson, & Williams, 2017; Ousterhout, Graham, Hasik, Serrano, & Siepielski, 2018; Start, 2018; Wood et al., 2020). Similarly, predator abundances, predator trait changes as well as changes in prey traits can modify predator–prey dynamics and abundances in numerous ways (Fryxell, Wood, Robinson, Kinnison, & Palkovacs, 2019; Griffiths, Petchey, Pennekamp, & Childs, 2018; de Roos et al., 2007; Walsh, DeLong, Hanley, & Post, 2012; Yamamichi & Miner, 2015). Therefore, understanding eco‐evolutionary dynamics in fisheries systems involves two interacting aspects: (a) In which ways fishing‐induced evolution affects the ecological dynamics of its food web? and (b) How natural selection arises from the altered ecological dynamics?

The existing ecosystem modelling tools have offered a straightforward way to study the first of the above questions. Namely, there exist whole‐ecosystem models, such as Atlantis (Audzijonyte, Gorton, Kaplan, & Fulton, 2017), developed for fisheries and environmental management purposes as well as more theoretical models such as the Allometric Trophic Network (ATN) model for community ecology analyses (Boit et al., 2012; Brose, Williams, & Martinez, 2006). Even though these models were not designed to study evolutionary processes, by forcing fish growth and age at maturity to decrease in an empirically defensible way (Audzijonyte, Kuparinen, & Fulton, 2013; Devine et al., 2012), the ecosystem models can provide some insights into the ecosystem‐wide long‐term consequences of contemporary fish life‐history changes.

As predicted by the Atlantis model, reduction in fish body size increases their vulnerability to predation and therefore increase their natural mortality (Audzijonyte, Kuparinen, Gorton, & Fulton, 2013), and lowers species’ carrying capacities such that recovery to virgin (prefishing) biomasses is not possible (Audzijonyte, Fulton, & Kuparinen, 2015). In general, the impacts of reduction in fish body sizes on other species in the ecosystem are comparable to those resulting from doubling the fishing pressure (Audzijonyte, Kuparinen, & Fulton, 2014). The ATN model parameterized for Lake Constance further suggested that reduction in fish body size can destabilize entire ecosystems (Kuparinen et al., 2016). Consistent with empirical mesocosm explorations (Bassar et al., 2010, 2012; El‐Sabaawi, Bassar, et al., 2015; El‐Sabaawi, Marshall, et al., 2015), the ecosystem modelling suggests that feedback of fish life‐history evolution to the ecosystem can be substantial. While the above‐mentioned studies focussed on evolutionary changes in fishes resulting from increased mortality, be it due to predation or fishing, it is notable that similar changes in fish phenotypes are predicted to occur because of increasing temperatures (Cheung et al., 2013). Thus, the projected consequences on the focal species itself and on the ecosystems are possibly generalizable to climate change scenarios (Audzijonyte et al., 2016; Audzijonyte & Waples, 2016).

## 4 DYNAMIC LIFE‐HISTORY EVOLUTION IN THE CONTEXT OF ECOSYSTEMS

### 4.1 Evolving food chains as the starting point

One early attempt to couple dynamic fishing‐induced evolution into multispecies interactions was performed by de Roos, Boukal, and Persson (2006), by using a consumer–resource model tuned for a fish exposed to selective harvesting and a prey species. The model encompassed mechanistic description of bioenergetic dynamics such that trade‐offs between life‐history traits and density‐dependent dynamics arise as emerging properties of the model dynamics (de Roos et al., 2006). Likewise, evolutionary changes in fish length at maturity evolved dynamically based on the assumed heritability and phenotypic variability within each cohort. Most importantly, the study by de Roos et al. (2006) demonstrated that the eco‐evolutionary consumer–resource dynamics can settle into alternative stable states. This means that eco‐evolutionary dynamics can be manifested through regime shifts in species life histories, which can be very difficult to reverse (de Roos et al., 2006). Indeed, it has been speculated that contemporary life‐history changes can affect the occurrence of tipping points and the ability of ecosystems to recover from regime shifts (Dakos et al., 2019).

Most models on fishing‐induced evolution focus on the evolution of targeted fish and its ecological consequences, while evolution of other interacting species as well as feedback loops through ecological processes back to selection driving evolution have merely been acknowledged as major omissions in previous approaches. A recent modelling study by Wood, Palkovacs, and Kinnison (2018) touches upon these limitations by using a four‐species food chain model that allows the producer and two consumers to evolve either one by one or simultaneously. The focus of this study was on the applied aspect of fishing, but it shifted the traditional focus from fishing impacts on the target top predator species to impacts mediated by ecological and evolutionary feedback loops through the lower trophic levels. In practice, the top predator was not allowed to evolve during simulated fishing, whereas the defence‐competition ability of the lower trophic levels could evolve. The study then investigated the impacts of this indirectly induced evolution coupled with direct ecological impacts on the top predator abundance and fishing yield (Wood et al., 2018).

In their four‐species food chain model, Wood et al. (2018) discovered that harvesting of the top predator cascades down the food chain both ecologically and evolutionarily. Harvesting caused changes in the abundances at each trophic level below the harvested species and increases in abundance lead to increased competitiveness, whereas decreases in abundance were associated with increased defence ability. If one trophic level was allowed to evolve at a time, the impact on the top predator abundance was either positive or negative, depending on the trophic distance of the predator from the evolving species. In a more realistic scenario that allowed the three lowest trophic levels to evolve simultaneously, the top predator abundance increased (Wood et al., 2018). While the findings might seem a relatively simple consequence of an abundance cascade in a top‐down controlled food chain, the outcome was further dependent on the trade‐off strength between the defence and the competitiveness as well as the fishing intensity. Thus, the study by Wood et al. (2018) suggests similar conclusions to those arising from the experimental guppy systems (Bassar et al., 2012): eco‐evolutionary multispecies dynamics is mediated through a complex set of direct and indirect mechanisms, making it impossible to intuitively predict the system‐level responses.

The major advance made by de Roos et al. (2006) and Wood et al. (2018) was that one or multiple species in the food chain evolved dynamically in response to changes in fishing coupled with species interactions. In other words, the rate and direction of evolution was not preset, as are many studies looking into fishing‐induced evolution in the ecosystem context (see the previous section). Instead, evolutionary responses emerged from the eco‐evolutionary feedback loops. Nonetheless, any food chain is still a very simplified description of the ecological complexity present in multispecies dynamics. In reality, a multispecies community consists of multiple partially overlapping food chains that together form a food web. Ecological dynamics alone are complex, and nonlinear in such webs and, from an evolutionary perspective, selection is likely to act on multiple, potentially correlated life‐history traits, which again feeds back to ecological dynamics.

### 4.2 Integration of fish life‐history evolution into a complex lake food web

Allometric trophic network models have recently provided a means to understand and predict the dynamics of complex food webs (Berlow et al., 2009; Brose et al., 2006). The idea of ATN models is that they base consumer–resource dynamics on empirically founded allometric scaling with body size (Brose et al., 2006). The ATN parameterization to Lake Constance in central Europe was the first study to apply the ATN models to an empirically well‐studied complex lake food web (Boit et al., 2012). ATN models with varying complexity were able to realistically simulate seasonal plankton biomass dynamics, suggesting that the ATN approach is able to generate reasonably realistic ecological multispecies dynamics (Boit et al., 2012).

Here, we build on this well‐studied complex lake food web and incorporate life‐history evolution into the ATN framework (hereafter, denoted as ATNE). We demonstrate the performance of the ATNE framework for Lake Constance by focussing on one fish species (Eurasian perch) and allowing its two life‐history traits to evolve dynamically and independently of each other. The traits in question are adult body size (i.e. the asymptotic size) and reproductive investment, both of which are known to readily evolve in response to increased mortality (e.g. Conover & Munch, 2002; Reznick et al., 1997; Uusi‐Heikkilä, Sävilammi, Leder, Arlinghaus, & Primmer, 2017; van Wijk et al., 2013).

#### 4.2.1 From ATN to ATNE

The ATNE model (Allometric Trophic Network with Evolution) is an evolutionary extension of the ATN model of food web dynamics, which allows for the evolution of fish life‐history traits. ATNE is built upon the ATN model developed by Kuparinen et al. (2016) which extended the ATN model by Boit et al. (2012) by introducing life‐history structure to the fish species in the food web (5 producers, 15 consumers and 5 fish life‐history stages for 2 fish species each, perch and whitefish). While in the ATN model of Kuparinen et al. (2016), the life‐history traits of the fishes were assumed constant, here the ATNE model assumes a distribution of fish life‐history traits which evolve based on natural selection arising from feeding interactions, and fishing‐induced selection.

The modelled food web contains feeding links among functionally distinct guilds of basal producers, consumers and fish life‐history stages (larvae, juveniles and a number of adult life‐history stages of different age fish). The dynamics of the system are divided into two parts. In the first part, the food web dynamics for year *Y* are simulated in continuous time during the “growth season.” This part includes producer species’ intrinsic growth, consumer and fish species’ feeding and maintenance of their bodily functions, and the allocation of portion of adult fish biomass for reproduction, as well as removal of adult fish biomass by fishing. These dynamics are modelled as a system of ordinary differential equations (ODEs). The second part of the system dynamics is called “reproduction and ageing,” and it consists of the birth of new fish larvae and the transfer of fish biomass to the next life stage for year *Y* + 1. The ecological dynamics of the food web as described by the ATN approach have been detailed by Kuparinen et al. (2016) and Kuparinen et al. (2019) so in the following we only focus on the components introduced to facilitate the life‐history evolution of fishes.

#### 4.2.2 Introduction of phenotypic variability

Each fish guild (i.e. fish life‐history stage) is split into “genotype groups,” and the *g*th genotype group of the guild *i* is denoted by ${\text{GG}}_{i,g}$. Each genotype group has a unique parameter vector $T_{i,g}$, which could in theory contain an arbitrary number of parameters. Here, we consider two parameters: the asymptotic body length of a fish $L_{i,g}^{\infty }$ and a parameter controlling the reproductive investment of mature fish $c_{i,g}^{R}$. Thus, $T_{i,g}=\left(L_{i,g}^{\infty },c_{i,g}^{R}\right)$. The asymptotic body length of a fish affects length‐at‐age, which allows for variability in metabolic rate. It also introduces variability in the maturation schedules of the fish (Charnov, 1993), thus affecting reproduction.

The genotype groups are defined by a grid in the trait parameter space. We set lower and upper limits for the trait parameters and set an equally spaced grid of $N=100^{2}=10,000$ grid cells in the parameter space. Each grid cell then represents a single genotype group. The parameter values for $T_{i,g}$ used in the model are the cell midpoints, and it is assumed that individuals within a genotype group are identical.

#### 4.2.3 Dynamic parameters for fishes

Unlike the traditional ATN approach, ATNE allows fish lengths to increase during the growth season and derives several other time‐varying parameters, such as the metabolic rate, based on the length at time point *t*.

The length of the fish, $L_{i,g}\left(t\right)$, is modelled with a von Bertalanffy growth model.

(1) $$L_{i,g}\left(t\right)=L_{i,g}^{\infty }‐\left(L_{i,g}^{\infty }‐L_{i}^{0}\right)e^{‐k_{i,g}\left(a_{i}+\frac{t}{t^{\text{end}}}\right)},$$

where $L_{i,g}^{\infty }$ is the asymptotic maximum length of a fish, $L_{i}^{0}$ is the fixed initial length of a fish larvae (at age $a_{i}=0$) at the beginning of the growth season (t=0), and $k_{i,g}$ is the growth rate parameter, which is negatively correlated with $L_{i,g}^{\infty }$ (Charnov, 1993). Here, we set $L_{i,g}^{\infty }$ to vary between 25 and 35 cm; $k_{i,g}$ was determined by fitting a von Bertalanffy growth curve to observed lengths at age for perch in Lake Constance (Kuparinen et al., 2016).

The metabolic rate of fish ${\text{GG}}_{i,g}$ is.

(2) $$x_{i,g}\left(t\right)=a_{x}{\left(\frac{M_{\text{ref}}}{M_{i,g}\left(t\right)}\right)}^{b_{x}},$$

where $a_{x}=0.88$ and $b_{x}=0.11$ are the allometric constant and scaling exponent for fish, respectively, $M_{\text{ref}}$ is the body mass of the reference guild (Boit et al., 2012; Kuparinen et al., 2016, 2019), $M_{i,g}\left(t\right)$ is the body mass of the fish ${\text{GG}}_{i,g}$ expressed as micrograms of carbon, which is calculated based on the fish length (Eqns. S1 and S2).

The proportion of mature biomass of fish ${\text{GG}}_{i,g}$ varies in time and across the genotype groups by linking it to the fish lengths and to one of the trait parameters, namely the asymptotic maximum length. We determine the proportion of mature biomass with sigmoidal maturity ogives as

(3) $$P_{i,g}\left(t\right)=\frac{1}{1+e^{\log\left(\frac{1‐p}{p}\right)\frac{L_{i,g}^{50\%}‐L_{i,g}\left(t\right)}{L_{i,g}^{50\%}‐L_{i,g}^{p}}}}.$$

The presented functional form of the maturity ogive uses three parameters: $L_{i,g}^{50\%}$ is the length at which half of the biomass is mature, $L_{i,g}^{p}$ is the length at which the fraction of mature biomass is 0<p<1. In our simulations, we set p=.05 and link these parameters to the asymptotic maximum length $L_{i,g}^{\infty }$ of the individuals in ${\text{GG}}_{i,g}$ as $L_{i,g}^{50\%}=\frac{2}{3}L_{i,g}^{\infty }$ and $L_{i,g}^{5\%}=\frac{1}{2}L_{i,g}^{\infty }$.

The fraction of mature surplus biomass that a given fish life‐history stage invests into reproduction is determined by the age *a_i_* in full years of the guild *i* and a genotype group‐specific trait value $c_{i,g}^{R}$, which was here set to vary between −0.04 and 0.04:

(4) $$I_{i,g}=\left\{\begin{array}{cc} 0.05a_{i}+c_{i,g}^{R}, & a_{i}\geq 2\\ 0, & a_{i}<2 \end{array}\right).$$

#### 4.2.4 Reproduction

**Figure 1:**
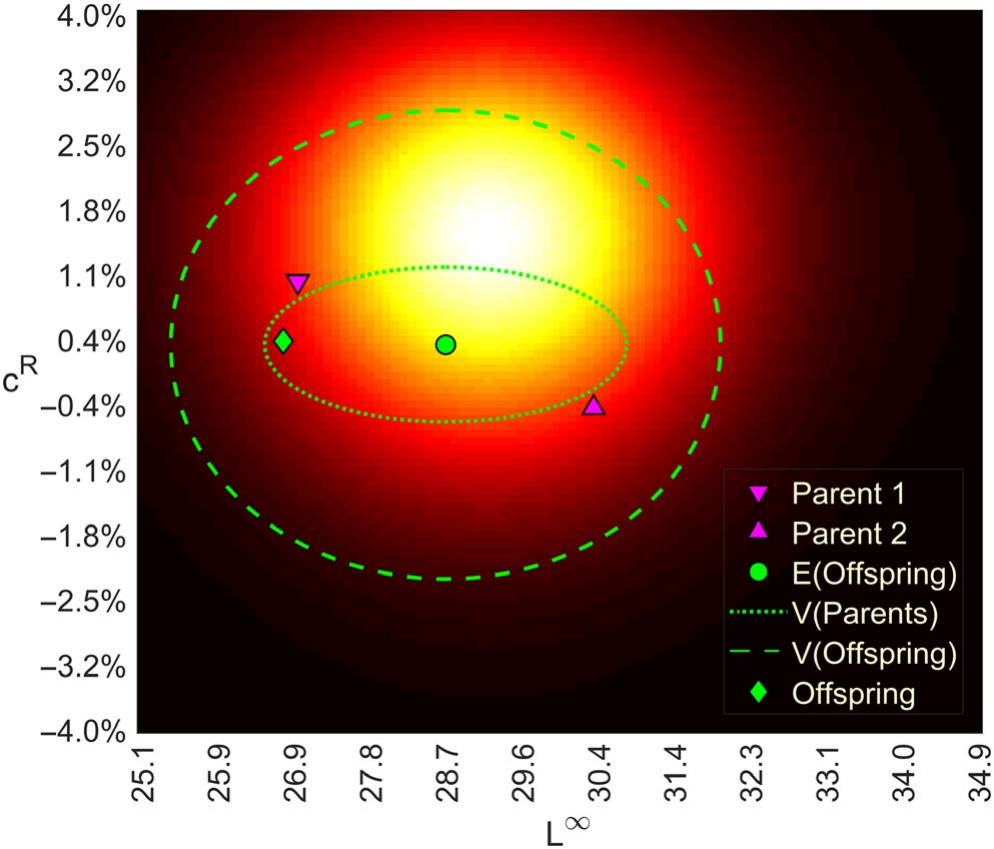
Illustration of the larvae trait distribution determination. The heat map shows the distribution of the traits in the reproductive output of the adult population, darker areas representing lower occurrence of the corresponding trait. The markings overlaying the heat map show the construction of the conditional probability distribution of the larvae trait value given a pair of parent trait values, which represents one component of the Gaussian mixture larvae trait distribution. The up and down pointing magenta triangles show one parent trait vector pair randomly drawn from the reproductive output. The green circle shows the mean of the conditional larvae trait distribution, and the dotted line ellipse shows the 95% containment probability region of the conditional trait distribution of the larvae without the phenotypic variance component (i.e. caused by the variation in the parent trait values), whereas the dashed line ellipse includes this variance component. The diamond shows a hypothetical larvae trait value drawn from this distribution and assigned to a grid cell

The reproductive output is calculated for each adult life‐history stage and for each ${\text{GG}}_{i,g}$ separately, and the reproductive outputs of each “genotype lineage” sum up the net reproductive outputs that have the same trait vector $T_{i,g}$. The resulting reproductive outputs are then divided by the total reproductive output of the fish species to obtain two identical discrete probability distributions of the parent trait values ($P(T^{F})$ and $P(T^{M})$) in the reproductive output. The conditional probability distribution of the larvae trait value, $T^{L}$, conditioned on a pair of parent trait values $\left(T^{F},T^{M}\right)$ is modelled as a multivariate normal distribution.

$$p\left(T^{L}|T^{F},T^{M}\right)=\text{MVN(}T^{L};\mu ,\Sigma ),$$

where the mean, $\mu =\text{mean(}T^{F},T^{M})$, is the average of the parent trait vectors, and its covariance matrix, $\Sigma =\text{diag(}c^{V}\text{var}\left(T^{F},T^{M}\right)+T^{A})$, is a diagonal matrix where the diagonal elements are composed of the sum of the variances of the parent trait values multiplied by a free parameter $c^{V}=0.125$, which controls the amount of variation caused by the parent trait values, and a parameter vector $T^{A}=\left(1,10^{‐4}\right)$, which accounts for phenotypic variation (Figure 1). These parameter values can be freely adjusted; here, the additive variance component was chosen so that heritability in the beginning of our simulations was approximately 30%, which is typical for quantitative life‐history traits (Mousseau & Roff, 1987). As the variability in the parent trait values change due to evolution and fishing during the simulation, so does the heritability. By using a diagonal covariance matrix, we assume no correlation between the traits. By the law of total probability and assuming independence between the trait values of the parents, the probability distribution of the larvae trait values can be written as.

$$p\left(T^{L}\right)=\sum_{T^{F}}\sum_{T^{M}}p\left(T^{L}|T^{F},T^{M}\right)P\left(T^{F}\right)P\left(T^{M}\right).$$

As we are using discrete probability distributions for the parent trait values, the larvae trait distribution is a mixture of multivariate normal distributions that are truncated to the grid in the trait space (Figure 1). Finally, we discretize the larvae trait distribution using the grid cells by simply calculating how much probability mass of the mixture distribution falls into each cell (i.e. genotype group). The grid boundaries represent upper and lower limits for the trait values in the simulation and are fixed beforehand.

#### 4.2.5 Food web dynamics

The total biomass of guild *i* and its derivative with respect to time are denoted by $B_{Y,i}\left(t\right)$ and ${\dot{B}}_{Y,i}\left(t\right)$, respectively, where $t\in [0,t^{\text{end}}]$, that is the start and the end of the growth season are t=0 and $t=t^{\text{end}}$, respectively. Here the length of the growth season was assumed to be $t^{\text{end}}=90$ days. The vector of all guild biomasses is denoted by $B_{Y}\left(t\right)$. Furthermore, for guilds with genotype group structure the biomass of the *g*th GG of guild i (${\text{GG}}_{i,g}$) and its derivative with respect to time are denoted by $B_{Y,i,g}\left(t\right)$ and ${\dot{B}}_{Y,i,g}\left(t\right)$, respectively. In this work, the genetic variability is considered only for fish species, but similar approach could be also used for the other species in the food web. We present the model equations for fish species using the genotype group notation.

The guild biomass dynamics consist of rates of biomass changes called *gains* (G) and *losses* (L) as summarized in Table 1. Below, we present the system of ODEs for all the different types of guilds accommodated by our model and refer to Supporting Information for the detailed formulation of each gain and loss component equation as well as the functional responses among the guilds.

**Table 1:** Gain and loss terms for each type of guild in the food web

	Gains		Losses			
Type	Intrinsic growth	Consumption	Maintenance	Consumption	Reproduction	Fishing
Equation	(S3)	(S4)	(S7, S8)	(S9, S10)	(S14)	(S12)
Producers	X			X		
Consumers		X	X	X		
Fish						
Larvae		X	X	X		
Juveniles		X	X	X		
Adults		X	X	X	X	X

The producer guild biomass dynamics consist of gains from their intrinsic growth and losses from feeding subjected to them by their herbivore predators. The time derivative of the biomass of the producer guild $i\in I_{P}$ is.

(5) $${\dot{B}}_{i}\left(t\right)=G_{i}^{\text{growth}}\left(t\right)‐\sum_{j\in I_{i}^{\text{predators}}}L_{i,j}^{\text{consumption}}\left(t\right)$$

The consumer guild dynamics consist of the maintenance of bodily functions, gains from feeding on their prey and losses due to getting fed on by their predators. The time derivative of the biomass of the consumer guild $i\in I_{C}$ is.

(6) $${\dot{B}}_{i}\left(t\right)=\left(\sum_{j\in I_{i}^{\text{prey}}}G_{i,j}^{\text{consumption}}\left(t\right)\right)‐L_{i}^{\text{maintenance}}\left(t\right)‐\left(\sum_{k\in I_{i}^{\text{predators}}}L_{i,k}^{\text{consumption}}\left(t\right)\right).$$

The fish guild dynamics are otherwise like the consumer guild dynamics, but they include additional losses due to fishing and reproduction. Furthermore, the fish dynamics are written for the “genotype groups.” The time derivative of the biomass of ${\text{GG}}_{i,g},i\in I_{F}$ is.

(7) $${\dot{B}}_{i,g}\left(t\right)=\left(\sum_{j\in I_{i}^{\text{prey}}}G_{i,g,j}^{\text{consumption}}\left(t\right)\right)‐L_{i,g}^{\text{maintenance}}\left(t\right)‐\left(\sum_{k\in I_{i}^{\text{predators}}}L_{i,g,k}^{\text{consumption}}\left(t\right)\right)‐L_{i,g}^{\text{fishing}}\left(t\right)‐L_{i,g}^{\text{reproduction}}\left(t\right).$$

Additionally, we want to solve the contribution of each fish “genotype group” to the next year's larvae production. We use ${\dot{B}}_{i,g}^{+}\left(t\right)$ to denote the time derivative of the contribution of fish ${\text{GG}}_{i,g}$ during the growth season, and add.

(8) $${\dot{B}}_{i,g}^{+}\left(t\right)=uL_{i,g}^{\text{reproduction}}\left(t\right)$$

to the system of ODEs, where u=0.8 is the efficiency with which the biomass allocated to reproduction is converted into larvae biomass (see Supporting Information for the reproduction loss Eqn. S14).

#### 4.2.6 Demonstration of ATNE framework in action

To illustrate how the above described ATNE framework functions, we utilize the fish life‐history structured food web for Lake Constance (Kuparinen et al., 2016, 2019). To this end, we first simulate food web dynamics in their dynamic equilibrium (400 years), followed by a period of selective fishing and a subsequent recovery period (each a 50‐year time period). We focus on the evolution of one fish species, the perch, with respect to two independently evolving traits: asymptotic body length and the reproductive investment parameter. The simulated fishing has a simple selectivity regime, either targeting fish below or above the selectivity threshold, which is either 15.9, 20.4 or 23.6 cm in length. These threshold values were chosen to roughly correspond the average body lengths of 2‐, 3‐, or 4‐year‐old perch, respectively. The yearly fishing mortality rate *E* was set to 0.5. Fishing did not directly target the reproductive investment parameter but, indirectly, as survival up to older ages is reduced owing to fishing, it may pay off to invest more to reproduction than to somatic growth. Selection on this trait thus arises from natural selection. The functional response parameters were determined according to the algorithm presented in Bland, Valdovinos, Hutchings, and Kuparinen (2019) where the fish larvae were treated as invertebrates, and otherwise the food web dynamics followed the parameterization described in Kuparinen et al. (2016, 2019; see Table S1) except for the values related to the implementation of evolution, which are detailed in the above model description.

**Figure 2:**
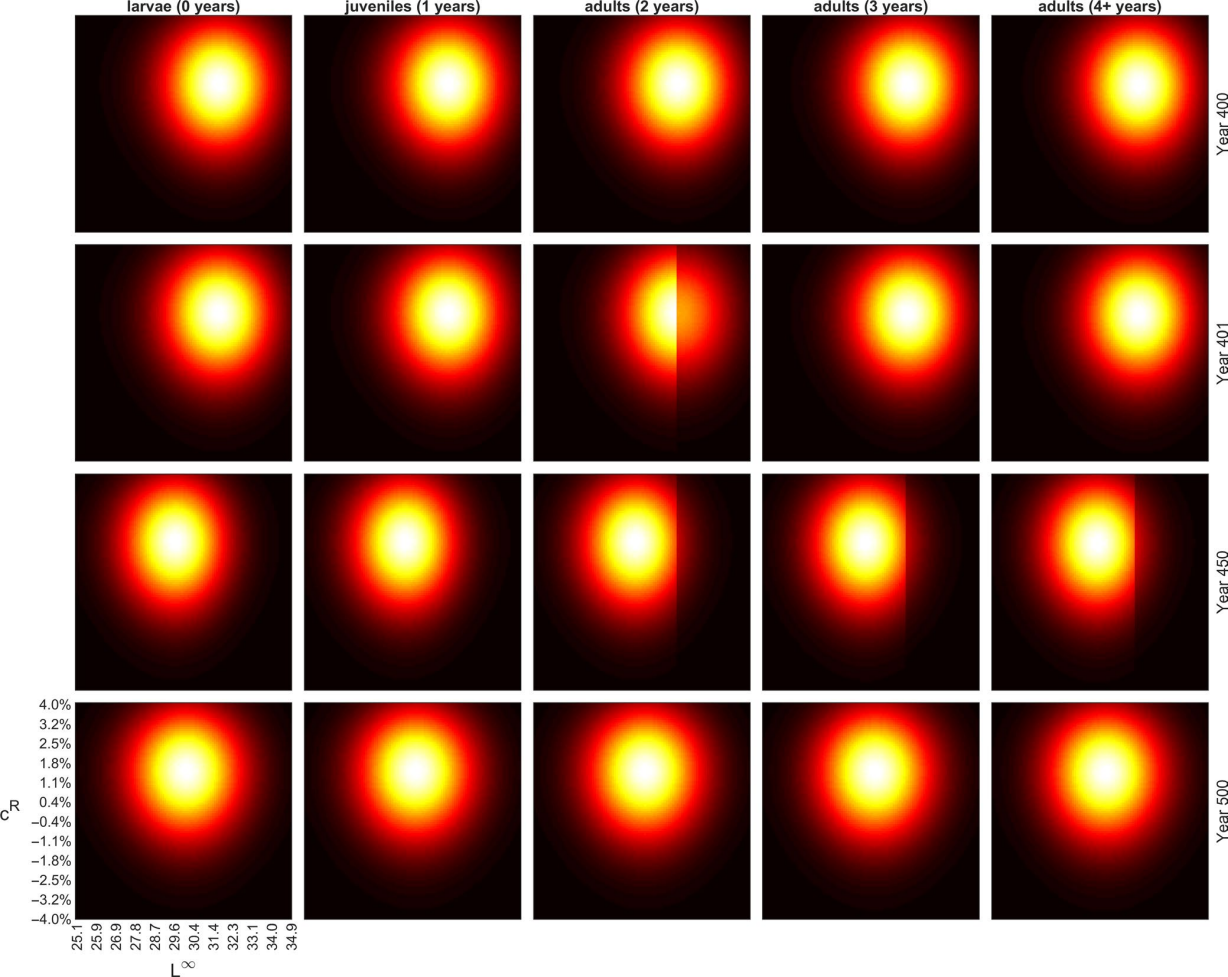
Evolution of the asymptotic maximum length, $L^{\infty }$, (horizontal axis) and reproductive investment parameter, $c^{R}$, (vertical axis) as fishing targets individuals larger than 15.9 cm. Snapshots of the simulated trait space are shown for four time steps: the last year before the fishing begins (Year 400), the first year of fishing (Year 401), the last year of fishing (Year 450) and the end of the recovery period (Year 500). See Figure 5 for the perch abundance development during the same simulated period; snapshot time steps are indicated in Figure 5 with grey triangles on the horizontal axis (time). Heat map colours illustrate the distribution varying from black (zero density) to red (low density) to white (high density). Fishing‐induced changes in the $L^{\infty }$ distribution of adult perches causes evolution in the species such that the trait values aggregate towards smaller $L^{\infty }$ values (see also Figure 4). As fishing ceases, natural selection arising from feeding interactions begins to push the traits towards their original distributions prior to fishing, but even after 50 years of recovery the change in $L^{\infty }$ is very small. For a demonstration of 150‐year (50‐year initialization, 50‐year fishing period, 50‐year recovery) simulation starting from a uniform trait distribution, see Video_Figure S2.avi in Supporting Information

**Figure 3:**
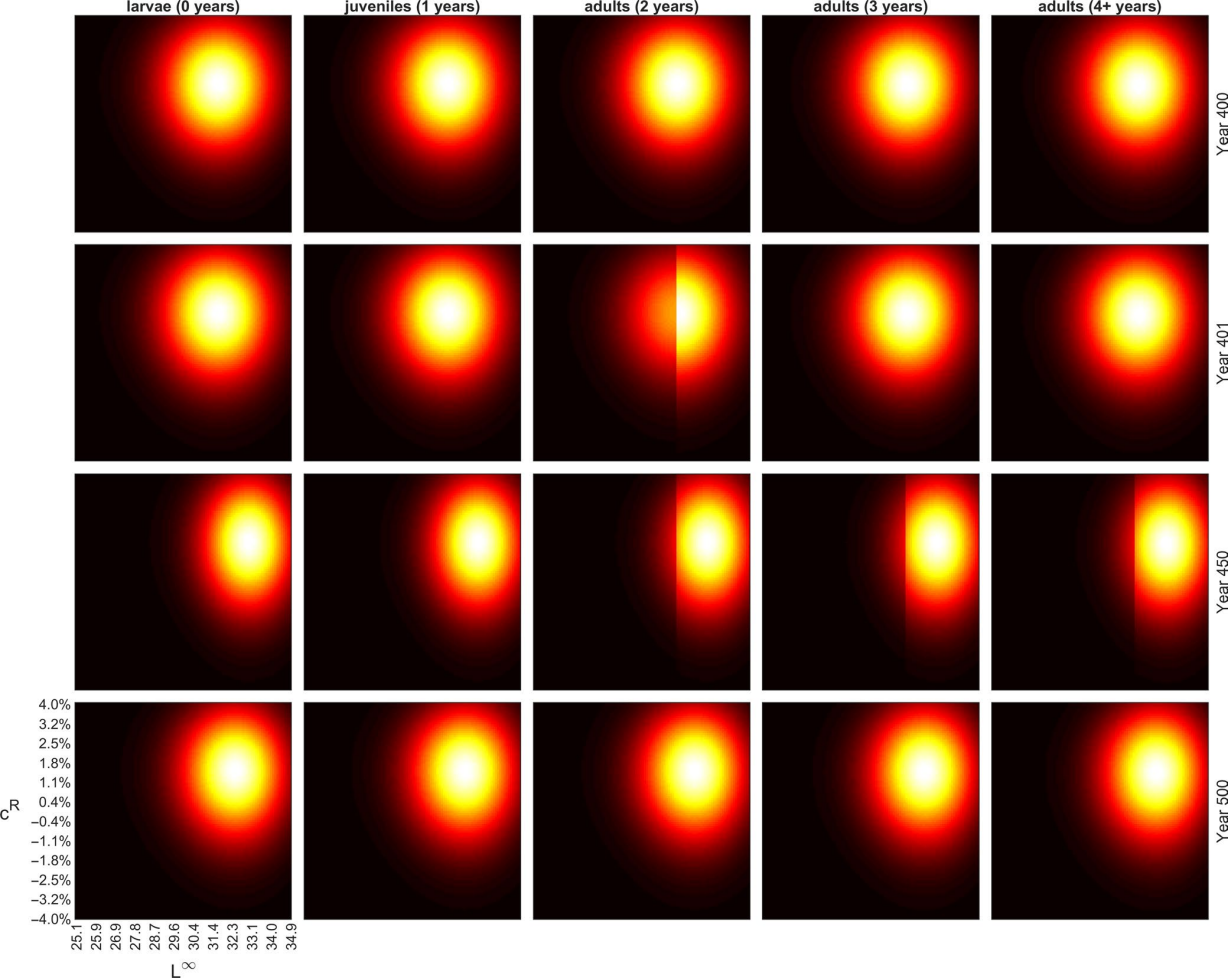
Evolution of $L^{\infty }$ (horizontal axis) and reproductive investment parameter (vertical axis) as fishing targets individuals smaller than 15.9 cm. Snapshots of the simulated trait space are shown for four time steps: the last year before the fishing begins (Year 400), the first year of fishing (Year 401), the last year of fishing (Year 450), and the end of the recovery period (Year 500). See Figure 5 for the perch abundance development during the same simulated period; snapshot points are indicated in Figure 5 with grey triangles on the vertical axis (time). Heat map colours illustrate the distribution varying from black (zero density) to red (low density) to white (high density). Fishing‐induced changes in the $L^{\infty }$ distribution of adult perches causes evolution in the species such that the trait values aggregate towards larger $L^{\infty }$ values (see also Figure 4). As fishing ceases, natural selection arising from feeding interactions begins to push the traits towards their original distributions prior to fishing, but even after 50 years of recovery the change in $L^{\infty }$ is very small. For a demonstration of 150‐year (50‐year initialization, 50‐year fishing period, 50‐year recovery) simulation starting from a uniform trait distribution, see Video_Figure S3.avi in Supporting Information

**Figure 4:**
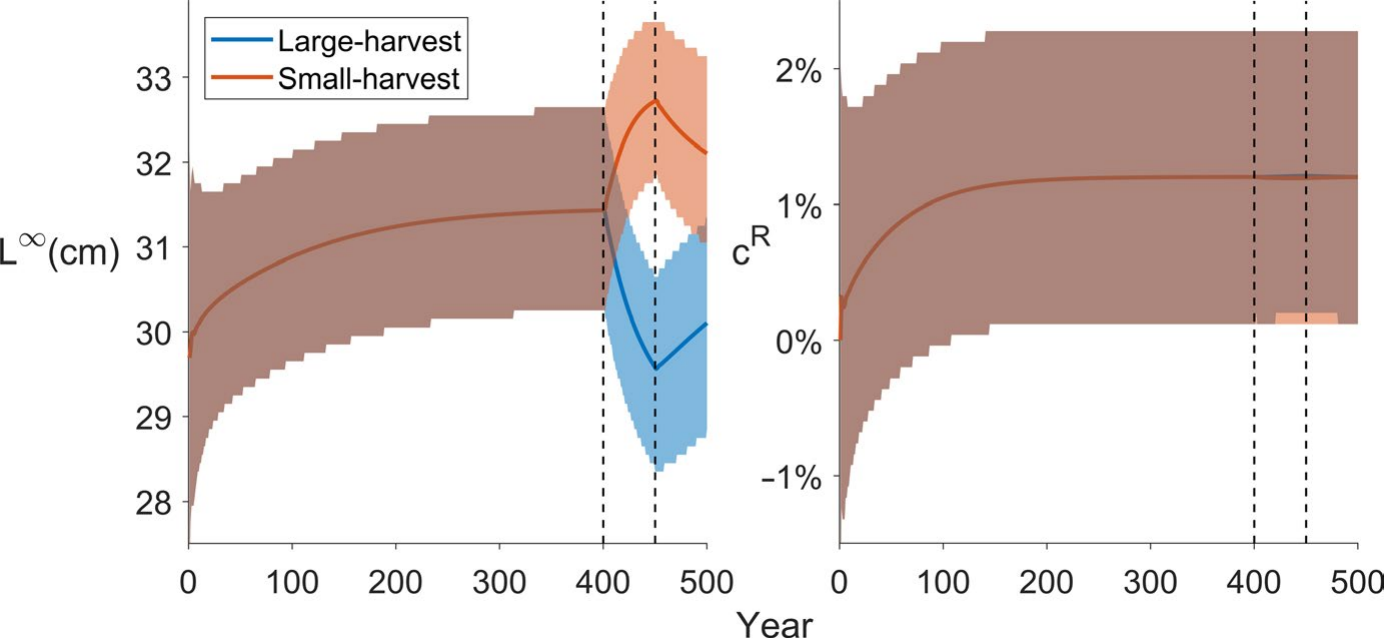
Evolution of perch larvae traits during the simulations. Blue (red) solid line shows the mean of the marginal distribution of the trait parameter in the population for the large‐harvest (small‐harvest) scenario. The shaded area represents the 50% central probability interval. The left and right panels represent the asymptotic maximum length ($L^{\infty }$) and the reproductive investment parameter ($c^{R}$), respectively

Temporal changes in the trait value grids are illustrated in Figures 2 and 3, for large‐ and small‐harvest scenarios with selection threshold at 15.9 cm, for each age‐class separately. For the other thresholds, the results are analogous and, thus, presented in Figures S1–S4. Figures 2 and 3 show snapshots of the progress of the simulation at four selected time points: (a) the last year before the onset of fishing (Year 400), (b) the first year of fishing (Year 401), (c) the last year of fishing (Year 450) and (d) the end of the recovery period (Year 500), whereas Figure 4 illustrates the changes in trait means and 50% central probability intervals plotted against time. The progress of simulations is also illustrated by videos available in Supporting Information; these demonstrate how the life‐history traits evolve dynamically from year‐to‐year.

The simulations were initiated with a uniform distribution of the trait values inside the grid space and within 400 years the trait values settle to their evolutionary equilibriums (driven by natural selection arising from consumer–resource dynamics; Figure 4). As fishing targets individuals larger than 15.9 cm, the abundances of the genotype groups with large asymptotic body lengths decrease in a knife‐edge manner among 2‐year‐old perch (Figure 2, Video1_Figure S2). Younger perch are not affected as their lengths are below the threshold and perch older than 2 years are larger than the threshold, so fishing selects all genotype groups similarly, thus preserving the initial $L^{\infty }$ distribution in the first year of fishing (Figure 2, 2nd row). As fishing continues, the 2‐year‐old perch truncated with respect to their $L^{\infty }$ distribution age and, thus, the truncation can also be seen in older age classes (Figure 2, 3rd row). Fishing‐induced changes in the $L^{\infty }$ distribution of adult perch guilds cause evolution in the species such that the trait values aggregate towards smaller $L^{\infty }$ values (Figure 2, 3rd row). As fishing ceases, natural selection arising from feeding interactions begins to push the traits towards their original distributions prior to fishing but 50 years of recovery is not sufficient to restore the original $L^{\infty }$ distribution (Figure 2, 4th row and Figure 4).

Fishing targeting individuals below the size threshold of 15.9 cm induces evolution towards larger $L^{\infty }$ values (Figure 3). The most notable difference between the small‐ and large‐harvest scenarios can be seen in the response to fishing and subsequent recovery: in the small‐harvest scenario, the change in $L^{\infty }$ distribution is smaller and, thus, it recovers closer to the original, whereas in large‐harvest scenario the change in $L^{\infty }$ is larger, and after the 50‐year recovery period, it is further away from the original as compared to the small‐harvest scenario (Figure 4). During the prefishing simulation period, the reproductive investment parameter evolves due to natural selection and reaches its evolutionary equilibrium. In both, the large‐ and the small‐harvest scenarios, changes in this parameter due to fishing are negligible, suggesting that this parameter is not selected by fishing.

**Figure 5:**
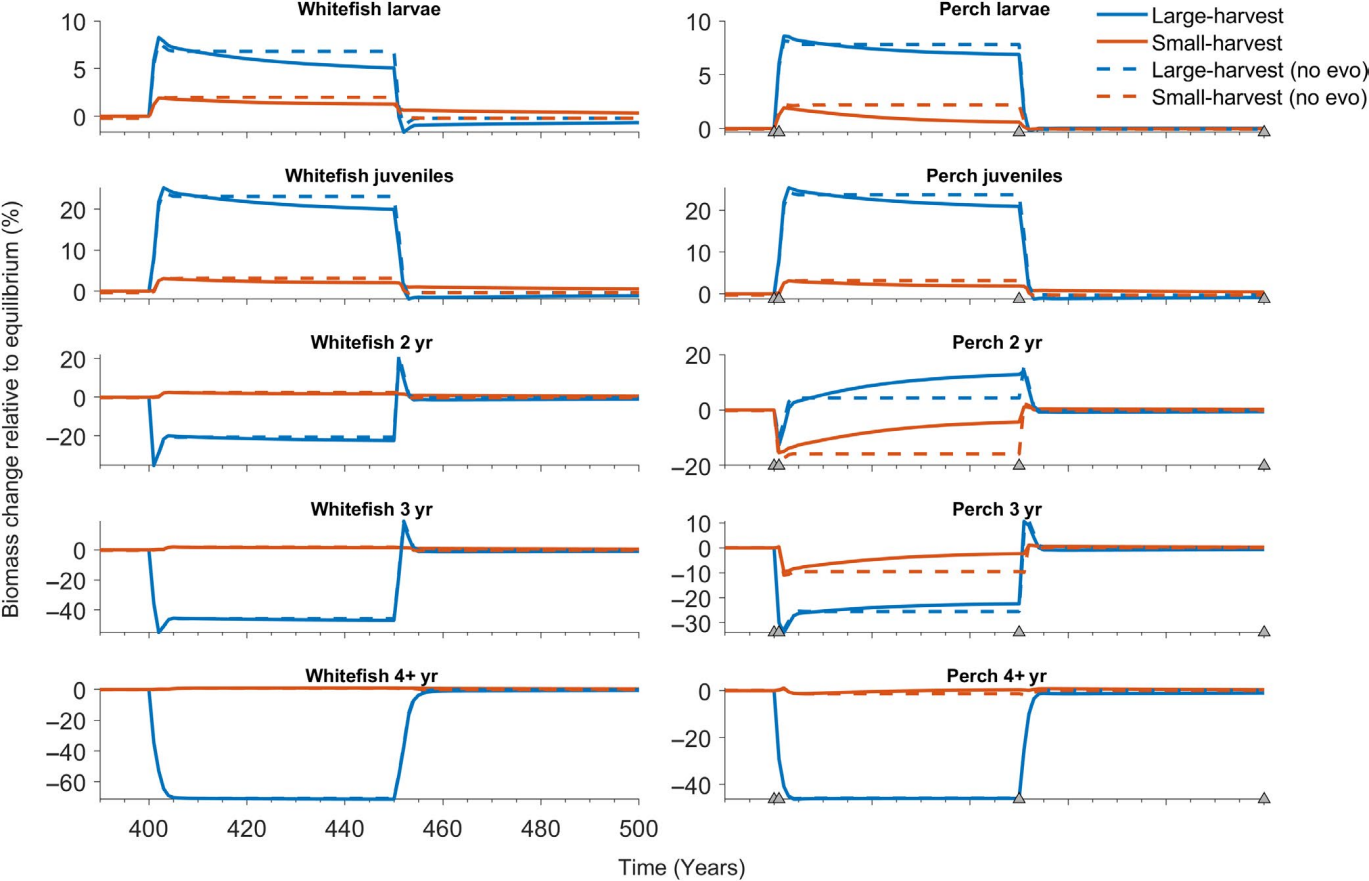
Biomass densities of fish guilds during four different scenarios. Blue (red) solid line shows the scenario where fishing targets individuals larger (smaller) than 15.9 cm. For comparison, the dashed lines represent scenarios where the perch larvae trait distribution remains constant throughout the simulation, and thus no evolution of perch life‐history traits occurs. The left‐ and right‐hand side columns show the five age classes of whitefish and perch, respectively. For perch, the grey triangles on the horizontal axis were added to denote the time steps used for the trait distribution snapshot in Figures 2 and 3 (years 400, 401, 450 and 500). The ecological effects of the perch life‐history evolution are more pronounced in the large‐harvest scenario. For the 2‐year‐old perch (Per2), large‐harvest reduces the average length of the fish and, thus, increases the average metabolic rate which in turn results in faster feeding and an increasing trend in the 2‐year‐old perch biomass density. This increased predator biomass and faster feeding can then be seen as a declining trend in the prey guilds of 2‐year‐old perch, namely fish larvae (Whi0 and Per0). Even though the ecological effects of fishing‐induced evolution are more visible during the fishing period (Years 401–450), there is also a difference in the biomass after the 50‐year recovery period indicating irreversible changes in the ecosystem biomass density distribution, especially for the large‐harvest scenario

**Figure 6:**
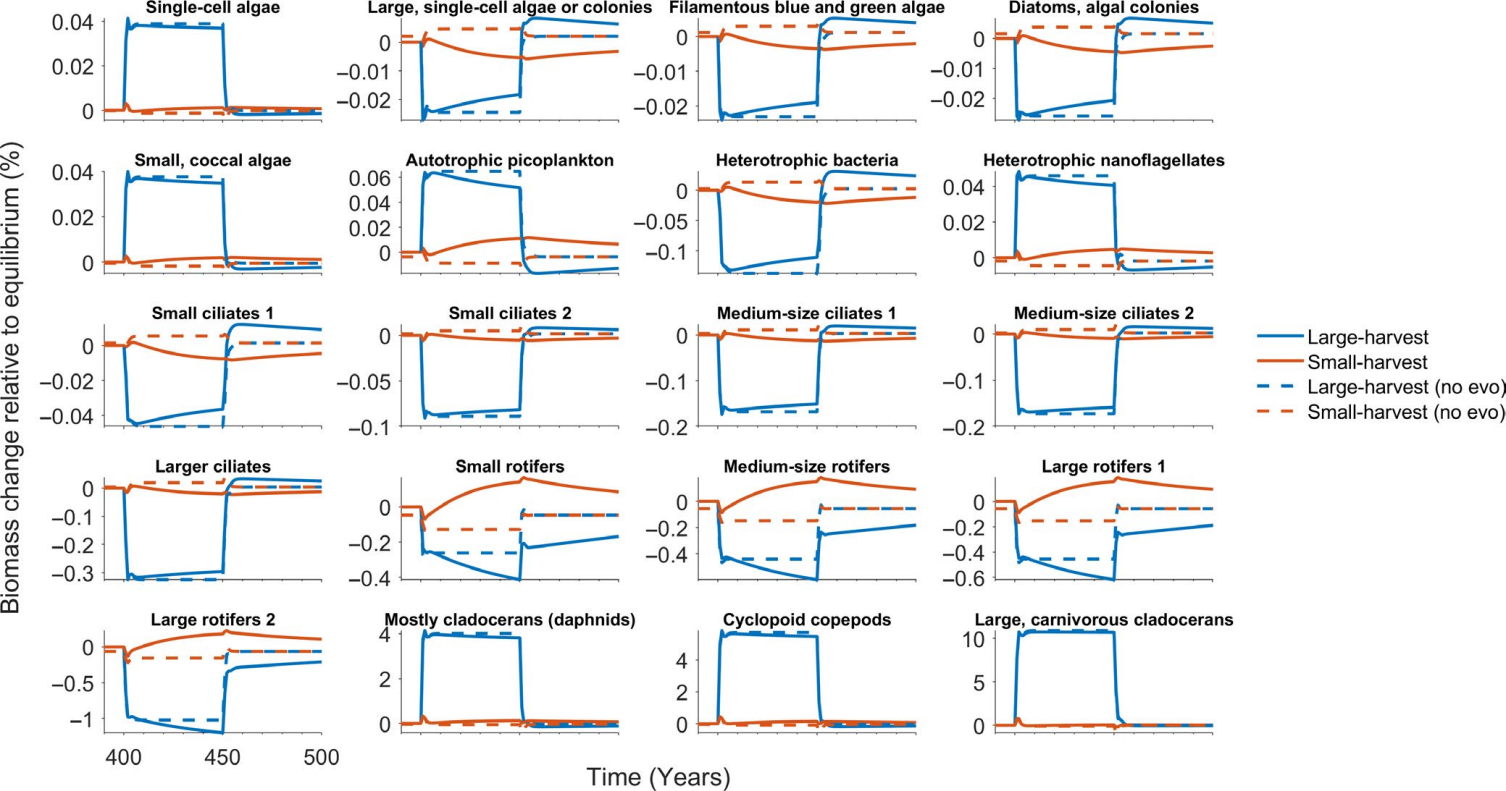
Biomass densities of producer and consumer guilds during four different scenarios. Blue (red) solid line shows the scenario where fishing targets individuals larger (smaller) than 15.9 cm. For comparison, the dashed lines represent scenarios where the perch larvae trait distribution remains constant throughout the simulation, and thus no evolution of perch life‐history traits occurs. The ecological effects of the life‐history evolution of the apex predator are visible on all trophic levels in the food web all the way down to the primary producers

As expected, fishing affected biomass abundances in the ecosystem but these ecological feedbacks were further modified by perch evolution and the harvesting scenario. Fish larvae and juvenile biomasses increased due to relaxed perch predation pressure (Figure 5) and increases in the abundances of key prey items (Figure 6). Large‐harvest scenario targeted older perch age classes and whitefish, whereas small‐harvest scenario reduced mainly younger adult perch (Figure 5). Changes in fish abundances were further affected by the evolution: for example, in the large‐harvest scenario the 2‐year‐old perch abundance increases towards the end of the fishing period as perch evolves to be smaller and, thus, its metabolic rate increases causing higher feeding rate while at the same time the guild becomes less targeted by harvesting. This feeds back to fish larvae, who experience greater predation pressure by adult perch (Figure 5), as well as further down to consumers and producers (Figure 6). It is notable that food web‐mediated feedback of perch evolution differs even within the same trophic level (e.g. among producers), underscoring the importance of a complex food web approach. As fishing is relaxed, the biomass levels into which the species recover are also affected by the evolution (Figures 5 and 6). The changes in perch guild equilibrium biomasses due to evolution are + 0.04% for larvae, −1.18% for juveniles, and −0.72%, −0.9% and −1.3% for 2‐, 3‐ and 4‐year‐old perch, respectively. In the small‐harvest scenario, the relative impact of perch evolution is less pronounced (larvae: −0.03%; juveniles: +0.75%, 2yr: +0.44%, 3yr: +0.55%; 4yr and older: +0.85%), because this fishing strategy does not truncate age‐class diversity in a similar manner.

#### 4.2.7 Advantages and limitations

The ATNE framework presented here provides means to simulate eco‐evolutionary dynamics in complex aquatic ecosystems, such as whole lake food webs, as was demonstrated here using Lake Constance as a case‐study. Evolutionary features were implemented to one species, as the other main fish species in Lake Constance is largely maintained by stocking (Kuparinen et al., 2016). However, the ATNE framework is generic, and the evolutionary features can be readily extended to several species, for more than two traits, and for different food webs. For fishes, this is particularly convenient due to life‐history invariants that link many fitness‐related life‐history traits to one parameter, the asymptotic maximum length, *L_∞_* (Charnov, 1993). For invertebrates, the major challenge is to come up with a set of traits most likely to respond to changes in predation pressure. One way to test this would be to model the evolution of competitiveness‐defence trade‐off as done by Wood et al. (2018). However, ultimately the traits in question depend on the study set‐up. If abiotic drivers are accounted for (see Boit et al., 2012), traits related to, for example, thermal tolerance might become relevant. The way genotype groups are implemented in the ATNE framework makes no assumptions about the nature of the trait and, as demonstrated in the present study, traits considered can be independent and affected by different drivers.

In its current formulation, ATNE does not incorporate any specific information about the genomic architecture of the traits but is based on the assumption that quantitative traits are coded by a large number of loci with small additive effects. However, nothing prevents from adding more weight to certain genotype groups mimicking loci with disproportionally large impacts on the phenotypes. This would require modifications in the way juvenile genotypes and phenotypes are assigned (Figure 1) and be specific for the genomic architecture desired.

## 5 FUTURE DIRECTIONS

In the near future, empirical experiments and sampling of natural populations are likely to suffer from similar limitations as today. However, computational power and numerical methods to simulate complex dynamics as well as investigations on genes coding fitness‐related traits are advancing at a rapid pace. While the former is necessary to solve the dynamics of increasingly complex systems, the latter is particularly important for understanding the ways in which traits can evolve. Particularly, so‐called supergenes with large impact on phenotypic traits (Barson et al., 2015; Thompson & Jiggins, 2014) can alter the nature of evolution in very unintuitive ways (Kuparinen & Hutchings, 2017). In order to ask the question of whether and how evolutionary changes in species affect the community and ecosystem dynamics, one needs to understand how traits are likely to evolve and change the phenotypes of the species. Nonetheless, the potential that genomics might offer to future studies of eco‐evolutionary dynamics of aquatic systems is not limited to trait architectures: genomics already today provide tools to identify ecologically meaningful units, that is populations and communities, as well as to estimate migration among populations (Bernatchez et al., 2017).

From an ecological point of view, there are still numerous unresolved issues related to the mechanisms and drivers of community dynamics. ATN and its evolutionary extension ATNE are convenient modelling frameworks as they utilize allometric scaling to estimate key parameters driving bioenergetic dynamics (Brose et al., 2006). In fishes, basic life‐history invariants are also useful for parameterizing life histories (Charnov, 1993), although it is notable that some species and populations might substantially differ from the invariants estimated across a range of species. Thus, invariants can serve as first proxies of trait correlations, but specific systems may require more detailed investigations of trait correlations and how those might change as evolution modifies phenotypes. Similarly, parameters determining functional responses among species are generally not well known and often set to system‐wide constants (e.g. Williams, 2008) or based on “educated guesses” (Bland et al., 2019). For example, predator preferences on different prey species are typically set equal (i.e. no preference for one over the other), yet the theory of adaptive foraging and even basic stomach content analyses suggest that predators do typically prefer certain prey items due to easier catchability and higher energetic or nutritional value. It is also a completely unexplored question to ask, whether the diet or the prey preferences might co‐evolve with other fitness‐related life‐history traits, such as adult body size. While functional responses can be studied, for example, in mesocosm settings, those are necessarily limited to few interacting species. In complex systems and natural populations, rapidly developing stable isotope and fatty acid analyses can shed light into the realized preferences in the future (Galloway et al., 2014; Nielsen, Clare, Hayden, Brett, & Kratina, 2018).

Taken together, the obvious future avenue for understanding eco‐evolutionary analyses in complex aquatic systems requires a synergistic approach that integrates experimental and modelling approaches with genomic and physiological techniques. The emergence of such a holistic perspective requires removal of boundaries among fields of biological research. While the study of eco‐evolutionary dynamics already seeks this by connecting ecological and evolutionary processes, the future of the field is to reach even further, on one hand to molecular and genomic techniques and, on the other hand, to behaviour, physiology and environmental research.

## CONFLICT OF INTEREST

None declared.

## AUTHOR CONTRIBUTIONS

AK designed the study and was responsible for the review part of the article and the conclusions. TP developed the codes, ran simulations and designed the illustrations. AK and TP interpret the results together. Both AK and TP contributed equally to the article.

## Supporting information

Supporting Information

## Data Availability

Codes and videos are available in Dryad: https://datadryad.org/stash/share/vDSHjV6JSbnX5JD0vhxr3UEZl6AMlkIRX56zwwOuawg
