# Supplementary material for: Eco‐evolutionary dynamics driven by fishing: From single species models to dynamic evolution within complex food webs
Source: Evol Appl. 2020 Aug 26;13(10):2507–20. doi: 10.1111/eva.13058 (PMC7691468; doi:10.1111/eva.13058)
Supplement: Supplementary file 1 — Supporting Information [file EVA-13-2507-s001.docx]

**Supplementary materials.**

**DYNAMIC WEIGHT-LENGTH CONVERSION FOR FISHES**

The body mass of the fish $\mathrm{GG}_{i,g}$ in micrograms of carbon is modelled as

| $M_{i,g}\left( t \right)=a_{M_{i}}c_{M_{i}}m_{i,g}\left( t \right),$ | (S1) |
| --- | --- |

where it is assumed that the dry weight of the fish is $c_{M_{i}}=0.2$ times its fresh weight, and that the amount of carbon is $a_{M_{i}}=0.53$ times the dry weight (Kuparinen et al. 2016). The fresh weight of the fish is modelled as

| $m_{i,g}\left( t \right)=a_{m_{i}}{L_{i,g}\left( t \right)}^{b_{m_{i}}},$ | (S2) |
| --- | --- |

where $a_{m_{i}}$ and $b_{m_{i}}$ are species-specific estimates of the length-weight relationship (Kuparinen et al. 2016). For perch $\left( a_{m_{i}},b_{m_{i}} \right)=\left( 0.0105, 3.11 \right)$ and for whitefish $(a_{m_{i}},b_{m_{i}})=(0.005, 3.14)$.

**GAINS**

**Intrinsic growth of producers.** The gain from the *intrinsic growth* of producer guild $i\in\mathcal{I}_{P}$ is modelled using a logistic growth model

| $\mathcal{G}_{i}^{\text{growth}}\left( t \right)=\left( 1-s_{i} \right)r_{i}B_{i}\left( t \right)\left( 1-\frac{1}{K}\sum_{j\in\mathcal{I}_{P}} c_{i,j}B_{j}\left( t \right) \right),$ | (S3) |
| --- | --- |

where $s_{i}$ is the fraction of exudation, $r_{i}$ is the mass-specific intrinsic growth rate, $c_{i,j}$ is a competition coefficient describing the strength of interspecific ($i\neq j$) and intraspecific ($i=j$) competition of shared resources, *K* is the parameter controlling (but not equal to) the
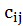
carrying capacity shared by all producer guilds, and $\mathcal{I}_{P}$ denotes the set of indices for the producer guilds (for parameter values, see Table S1).

**Consumption.** The gain for guild $i$ from the *consumption* of its prey guild $j\in\mathcal{I}_{i}^{\text{prey}}$ is

| $\mathcal{G}_{i,j}^{\text{consumption}}\left( t \right)=f_{a}x_{i}B_{i}\left( t \right)y_{i,j}F_{i,j}\left( \boldsymbol{B}\left( t \right) \right),$ | (S4) |
| --- | --- |

where $f_{a}$ is the activity maintenance cost, $x_{i}$ is the metabolic rate, $y_{i,j}$ is the maximum consumption rate of guild $i$ feeding on guild $j$, and

| $F_{i,j}\left( \boldsymbol{B}\left( t \right) \right)=\frac{\omega_{i,j}{B_{j}\left( t \right)}^{q}}{{B0}_{i,j}^{q}+d_{i,j}{B0}_{i,j}^{q}B_{i}\left( t \right)+\sum_{k\in\mathcal{I}_{i}^{\text{prey}}} \omega_{i,k}{B_{k}\left( t \right)}^{q}}$ | (S5) |
| --- | --- |

is the predator guild’s normalized functional response to its prey species densities, where $\omega_{i,j}$ is the predator’s relative prey preference, $q=1.2$ is the Hill’s exponent which forms a relatively stable version of the Holling Type-II functional response (Williams & Martinez 2008), ${B0}_{i,j}$ is the half saturation constant describing the biomass of the prey at which the predator achieves half of its maximum feeding rate when consuming only prey $j$ and in the absence of feeding interference, $d_{i,j}$ is the coefficient of intraspecific feeding interference.

The fish guilds have time-varying metabolic rates, and the genotype group structure. The consumption gains are calculated for each $\text{GG}_{i,g}$ separately as

| $\mathcal{G}_{i,g,j}^{\text{consumption}}\left( t \right)=f_{a}x_{i,g}\left( t \right)B_{i,g}\left( t \right)y_{i,j}F_{i,j}\left( \boldsymbol{B}\left( t \right) \right)$ | (S6) |
| --- | --- |

**LOSSES**

**Maintenance of bodily functions.** The loss due to *maintenance* of bodily functions is

| $\mathcal{L}_{i}^{\text{maintenance}}\left( t \right)=f_{m}x_{i}B_{i}\left( t \right)$ | (S7) |
| --- | --- |

where $f_{m}$ is the fraction of biomass respired by maintenance of basic bodily functions. This parameter is increased by 100% for the mature individuals to create the trade-off between current and future reproduction, i.e. the survival cost of reproduction (Bell 1980). For $\text{GG}_{i,g}$

| $\mathcal{L}_{i,g}^{\text{maintenance}}\left( t \right)=f_{m}x_{i,g}\left( t \right)B_{i,g}\left( t \right)$ | (S8) |
| --- | --- |

**Consumption.** The *consumption* loss for guild $i$ due to getting fed on by its predator guild $j$ is

| $\mathcal{L}_{i,j}^{\text{consumption}}\left( t \right)=\frac{1}{e_{j,i}}\mathcal{G}_{j,i}^{\text{consumption}}\left( t \right)$ | (S9) |
| --- | --- |

where $e_{j,i}$ is the assimilation efficiency describing the fraction of ingested biomass lost by egestion. For $\text{GG}_{i,g}$ we distribute the total loss of guild $i$ based on the distribution of the guild biomass and induce greater losses to smaller individuals.

| $\mathcal{L}_{i,g,j}^{\text{consumption}}\left( t \right)=\pi_{i,g}(t)\mathcal{L}_{i,j}^{\text{consumption}}\left( t \right)$ | (S10) |
| --- | --- |

Where the fraction of the total loss assigned to $\text{GG}_{i,g}$ is proportional to its biomass and suitably chosen function of its length

| $\pi_{i,g}(t)\propto B_{i,g}\left( t \right)\cdot{L_{i,g}\left( t \right)}^{-\frac{1}{b_{m_{i}}}}.$ | (S11) |
| --- | --- |

**Fishing**. The loss for $\text{GG}_{i,g}$ caused by fishing using a size selective fishing gear is modelled as

| $\mathcal{L}_{i,g}^{\text{fishing}}\left( t \right)=h_{i,g}\left( t \right)B_{i,g}\left( t \right),$ | (S12) |
| --- | --- |

where $h_{i,g}\left( t \right)=ES_{i,g}\left( t \right)$ is the daily fishing mortality of genotype group $g$ of guild $i$ at time $t$ when the fishing effort is $E=H/t^{\mathrm{end}}=0.5/90$ and the selectivity of the fishing gear for genotype group $g$ of guild $i$ at time t is $S_{i,g}\left( t \right)$. In our simulations we use fishing gears that target only adult fish ($a_{i}\geq2)$ genotype groups whose body lengths $L_{i,g}\left( t \right)$ are below (*small selected*) or above (*large selected*) a certain cutoff length $L^{c}$, i.e.,

| $S_{i,g}^{\text{small selected}}\left( t \right)=\left\{ \begin{matrix} 1, & L_{i,g}\left( t \right)\leq L^{c}\wedge a_{i}\geq2 \\ 0, & \text{otherwise} \end{matrix} \right.$  $S_{i,g}^{\text{large selected}}\left( t \right)=\left\{ \begin{matrix} 1, & L_{i,g}\left( t \right)\geq L^{c}\wedge a_{i}\geq2 \\ 0, & \text{otherwise} \end{matrix} \right.$ | (S13) |
| --- | --- |

**Fish reproduction.** The loss for $\text{GG}_{i,g}$ caused by allocating biomass for *reproduction* is

| $\mathcal{L}_{i,g}^{\text{reproduction}}\left( t \right)=\left\{ \begin{matrix} P_{i,g}\left( t \right)I_{i,g}\cdot\frac{{\mathcal{G}_{i,g}\left( t \right)}^{2}}{2\mathcal{L}_{i,g}\left( t \right)}, & \mathcal{G}_{i,g}\left( t \right)<\mathcal{L}_{i,g}\left( t \right) \\ P_{i,g}\left( t \right)I_{i,g}\cdot\left( \mathcal{G}_{i,g}\left( t \right)-\frac{1}{2}\mathcal{L}_{i,g}\left( t \right) \right), & \mathcal{G}_{i,g}\left( t \right)\geq\mathcal{L}_{i,g}\left( t \right) \end{matrix} \right.$ | (S14) |
| --- | --- |

where $P_{i,g}\left( t \right)$ denotes the proportion of mature biomass in $\text{GG}_{i,g}$ at time $t$ (Eqn. 2), and $I_{i,g}$ denotes the fraction of the mature surplus biomass that is invested into reproduction (Eqn. 3). This piecewise defined model is used to enforce impaired reproduction when the maintenance losses are greater than the consumption gains. The reproduction losses depend on the total gain for fish $\text{GG}_{i,g}$ which is the sum of the consumption gains over all of its prey species

| $\mathcal{G}_{i,g}\left( t \right)=\sum_{j\in\mathcal{I}_{i}^{\text{prey}}} \mathcal{G}_{i,g,j}^{\text{consumption}}\left( t \right)$ | (S15) |
| --- | --- |

and on the total loss without the reproduction which is the sum of maintenance, fishing and consumption losses to each predator

| $\mathcal{L}_{i,g}\left( t \right)=\mathcal{L}_{i,g}^{\text{maintenance}}\left( t \right)-\left( \sum_{k\in\mathcal{I}_{i}^{\text{predators}}} \mathcal{L}_{i,g,k}^{\text{consumption}}\left( t \right) \right)-\mathcal{L}_{i,g}^{\text{fishing}}\left( t \right).$ | (S16) |
| --- | --- |

**Table 1** Summary of the ATN model parameters for Lake Constance. Adapted from Kuparinen et al. (2016, 2018).

| **Parameter** | **Unit** | **Value** | **Description** | **Reference** |
| --- | --- | --- | --- | --- |
| *K* | μgC/m^3^ | 540000 | Phytoplankton carrying capacity coefficient | Boit et al. 2012 |
| *x_i_* | 1/day | 0.04 – 0.43 | Mass-specific metabolic rate^1^ | Brose et al. 2006 |
| *r_i_* | 1/day | 0.6 – 1.2 | Mass-specific growth rate for autotrophs^1^ | Brose et al. 2006 |
| *c_ij_* |  | 1 (2 for i=j) | Producer competition coefficient | Boit et al. 2012 |
| *f_a_* |  | 0.4 | Activity metabolism coefficient | Humphreys 1979 |
| *f_m_* |  | 0.1 | Maintenance respiration coefficient | Humphreys 1979 |
| *y_ij_* |  | 10 | Maximum ingestion rate | Brose et al. 2006, Yodzis & Innes 1992 |
| *e_ij_* |  | 0.45 – 1 | Assimilation efficiency | Brose et al. 2006 |
| *d_ij_* | m^3^/μgC | 0 – 1 | Feeding interference coefficient | Bland et al. 2019 |
| *q* |  | 1.2 | Functional response shape parameter | Boit et al. 2012 |
| *ω_ij_* |  | 0 – 0.5 | relative prey preference | Boit et al. 2012 |
| *s_i_* |  | 0.2 | fraction of exudation | Boit et al. 2012 |
| *B0_ij_* | μgC/m^3^ | 1500 – 150000 | Half-saturation densities | Bland et al. 2019 |

^1^ Except for fishes for which this parameter changes dynamically. The rates are relative with respect to guild 1.

**References**

Bell, G. (1980) The costs of reproduction and their consequences. *Am. Nat.* 116, 45–76.

Bland, S., Valdovinos, F.S., Hutchings, J.A., & Kuparinen, A. (2019) The role of fish life histories in allometrically scaled food‐web dynamics. *Ecology and evolution*, 9, 3651–3660.

Boit, A., Martinez, M.D., Williams, R.J & Gaedke, U. (2012) Mechanistic theory and modeling of complex food web dynamics in Lake Constance. *Ecol. Lett.* 15, 594–602.

Brose, U., Williams, R.J. & Martinez, N.D. (2006) Allometric scaling enhances stability in complex food webs. *Ecol. Lett.* 9, 1228–1236.

Humphreys, W. F. (1979) Production and respiration in animal populations. *J. Anim. Ecol.* 48: 427–453.

Kuparinen, A., Boit, A., Valdovinos, F.S., Lassaux, H. & Martinez, N.D. (2016) Fishing-induced life-history changes degrade and destabilize harvested ecosystems. *Sci. Rep.* 6, 22245.

Kuparinen, A., Perälä, T., Martinez, N.D.M. & Valdovinos, F.S. (2019) Environmentally-induced noise dampens and reddens with increasing trophic level in a complex food web. *Oikos* 128, 608-620.

Nielsen, M. V. & Olsen, Y. (1989) The dependence of the assimilation efficiency in Daphnia magna on the 14C-labeling period of the food algae Scenedesmus acutus. *Limnol. Oceanogr.* 34, 1311–1315.

Skalski, G. T. & Gilliam, J. F. (2001) Functional responses with predator interference: viable alternatives to the Holling type II model. – Ecology 82, 3083–3092.

Williams, R.J. & Martinez, N.D. (2008) Success and its limits among structural models of complex food webs. *J. Anim. Ecol.* 77, 512–519.

Yodzis, P. & Innes, S. (1992) Body size and consumer-resource dynamics. *Am. Nat.* 139, 1151–1175.

**Supplementary videos**

Videos appended to the supplementary materials illustrate how simulations progress at a year-by-year basis. Videos are named according to corresponding figures, although the pre-fishing simulation time has been truncated in the videos due to visualization purposes. The videos can be found at: <https://datadryad.org/stash/share/vDSHjV6JSbnX5JD0vhxr3UEZl6AMlkIRX56zwwOuawg>.

**Video_Fig2.avi**

**Video_Fig3.avi**

**Video_FigS1.avi**

**Video_FigS2.avi**

**Video_FigS3.avi**

**Video_FigS4.avi**


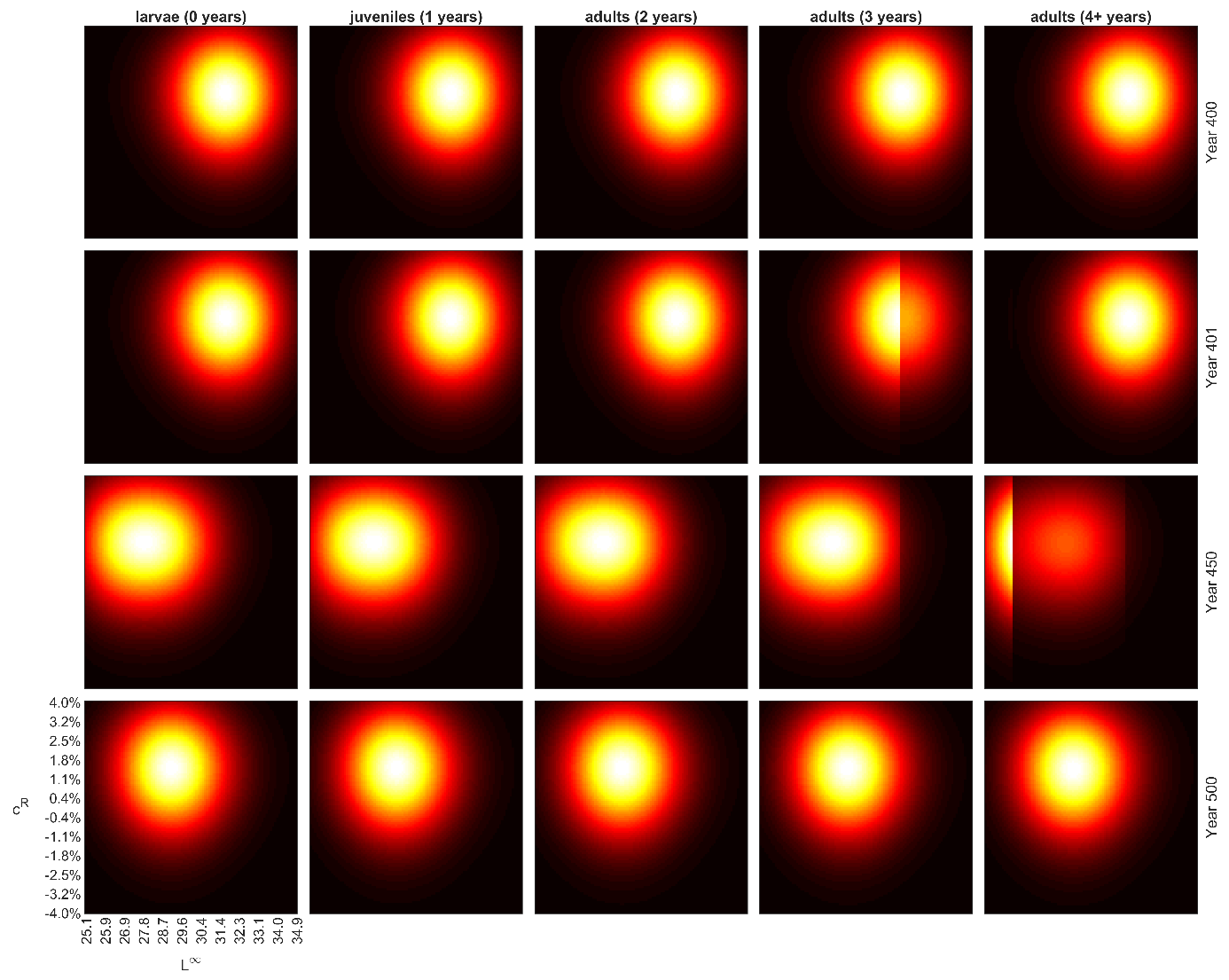
**Fig. S1** Large-harvest simulation with selection threshold 20.4 cm. All the figure elements correspond to those in Figs. 2 and 3.


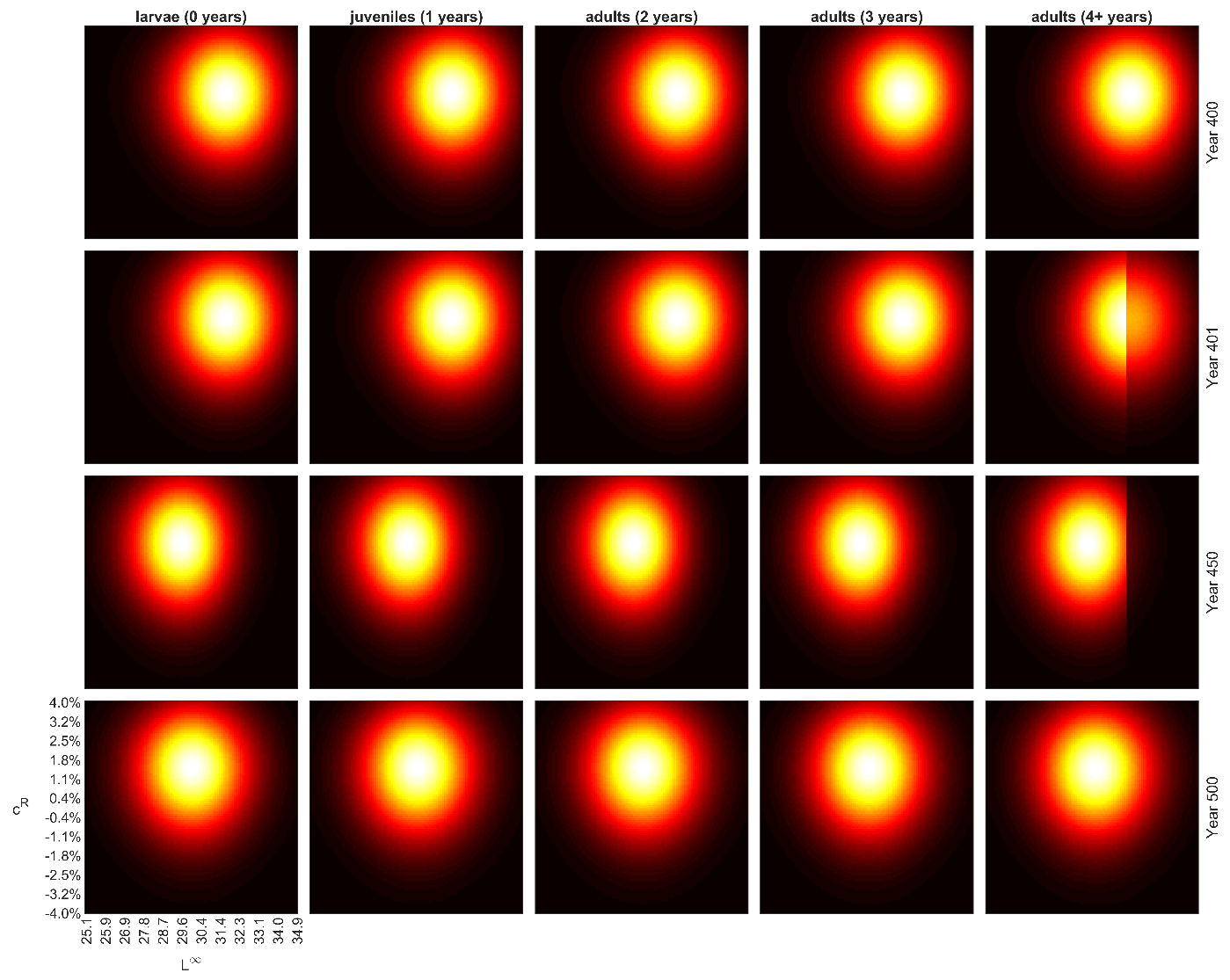
**Fig. S2** Large-harvest simulation with selection threshold 23.6 cm. All the figure elements correspond to those in Figs. 2 and 3.


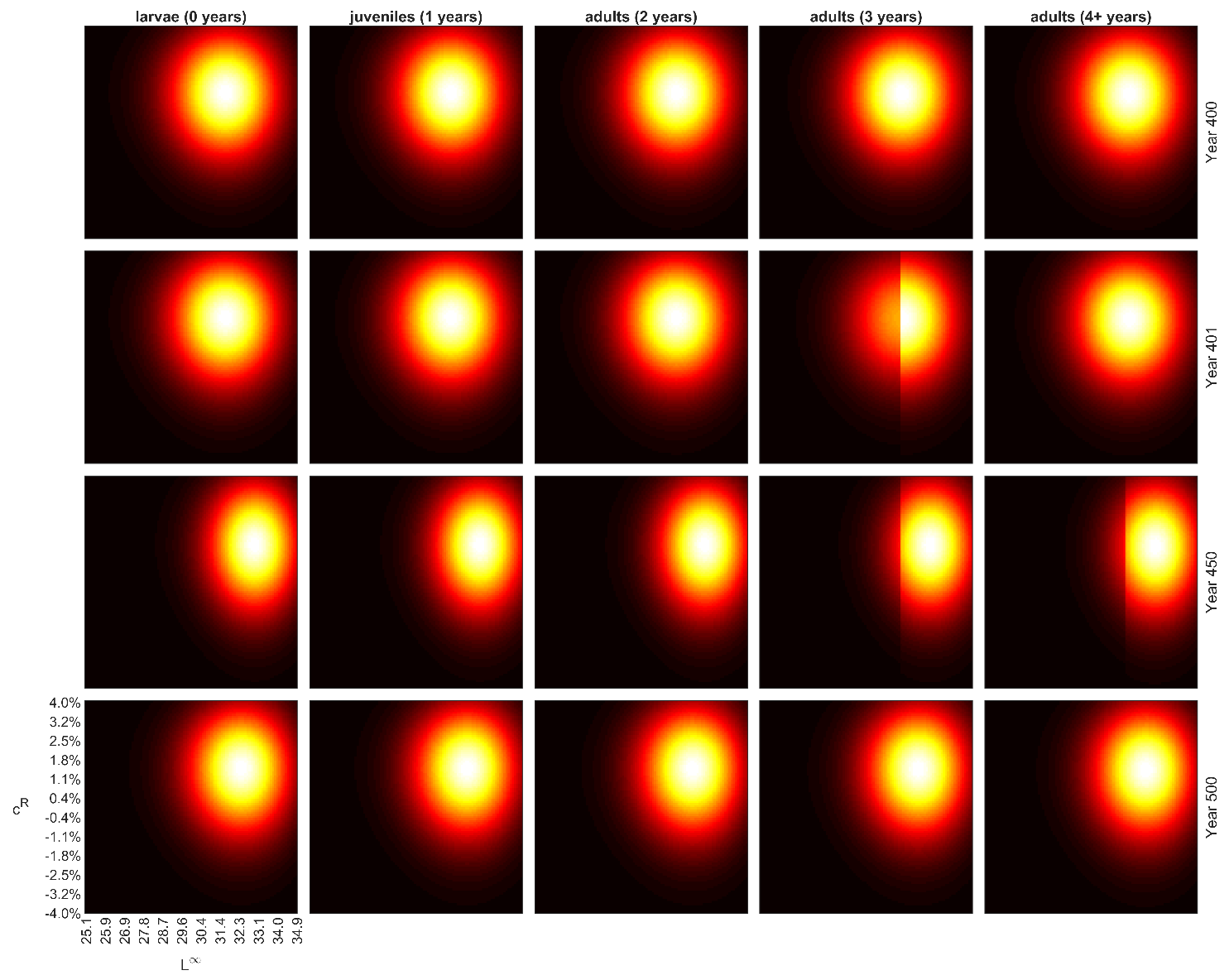
**Fig. S3** Small-harvest simulation with selection threshold 20.4 cm. All the figure elements correspond to those in Figs. 2 and 3.


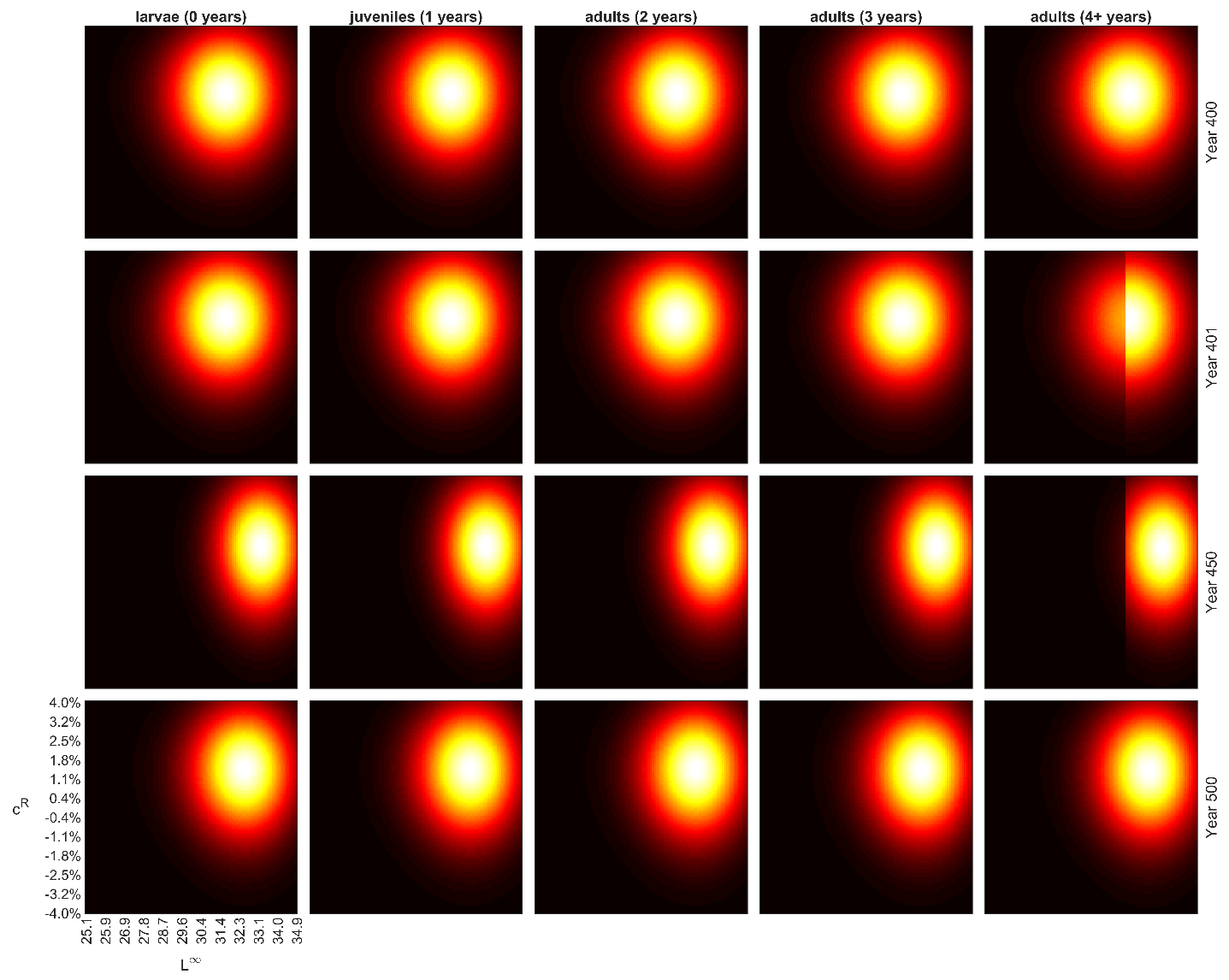
**Fig. S4** Small-harvest simulation with selection threshold 23.6 cm. All the figure elements correspond to those in Figs. 2 and 3.
